# Intersubject Synchronization of Late Adolescent Brain Responses to Violent Movies: A Virtue-Ethics Approach

**DOI:** 10.3389/fnbeh.2019.00260

**Published:** 2019-11-22

**Authors:** Azeez Adebimpe, Danielle S. Bassett, Patrick E. Jamieson, Daniel Romer

**Affiliations:** ^1^Annenberg Public Policy Center, University of Pennsylvania, Philadelphia, PA, United States; ^2^Department of Psychiatry, Perelman School of Medicine, University of Pennsylvania, Philadelphia, PA, United States; ^3^Department of Bioengineering, School of Engineering and Applied Science, University of Pennsylvania, Philadelphia, PA, United States; ^4^Department of Electrical and Systems Engineering, School of Engineering and Applied Science, University of Pennsylvania, Philadelphia, PA, United States

**Keywords:** fMRI, violent movies, moral judgment, brain synchronization, virtue-ethics

## Abstract

Movies that involve violence increasingly attract large audiences, leading to concern that such entertainment will encourage imitation by youth, especially when the violence is seen as justified. To assess differences between brain responses to justified and unjustified film violence, we computed intersubject correlation (ISC) of fMRI BOLD time courses in a sample of late adolescents while they watched pairs of movie segments featuring violent characters prior to and during violent action. Based on a virtue-ethics approach that emphasizes motives in moral evaluation, we hypothesized significant ISC in lateral orbital frontal cortex (lOFC) and ventromedial prefrontal cortex (vmPFC) in response to unjustified and justified scenes of movie violence, respectively. Our predictions were confirmed. In addition, unjustified violence elicited greater intersubject synchrony in insular cortex, consistent with an empathic response to the pain experienced by victims of this kind of violence. The results provide evidence supporting the notion that lOFC and vmPFC play unique roles in moral evaluation of violence, with lOFC becoming more synchronous in response to unacceptable violence and vmPFC becoming more synchronous in response to virtuous forms of self-defense, thereby expanding the purview of current models that only focus on vmPFC. The results suggest that justified violence in popular movies is acceptable to youth who are accustomed to viewing such entertainment.

## Introduction

Movies are a popular form of entertainment that not only hold people’s attention but also provide models of behavior (Bandura, 2001; Bushman and Huesmann, 2006). Years of research have found reliable evidence of the socializing effects of repeated exposure to violence in entertainment media and aggressive tendencies in youth (Anderson et al., 2017). However, some portrayals are more likely to be emulated than others. Violence that is seen as justified, in which characters kill others in self-defense or to protect friends and family are more likely to register approval than violence that has no socially redeeming value (Samson and Potter, 2016; Romer et al., 2018). Furthermore, laboratory studies have found that exposure to justified film violence reduces inhibitions to act aggressively to a greater degree than exposure to unjustified violence (Berkowitz, 1984). Here we investigate whether different brain mechanisms underlie responses to these two types of violence as portrayed in popular movies. We also compare competing neuroscience models of moral evaluation regarding their predictions for responses to these different forms of violence.

Most research regarding the neural underpinnings of moral evaluation focuses on dilemmas that pose a conflict between following rigid moral norms such as “do not kill” and more flexible utilitarian approaches that allow killing if it saves more lives in the process. The most famous of these dilemmas involves various versions of the trolley problem where one is asked to decide if it is justified to kill one person in order to save several others. In the various models that have been proposed to explain brain responses to these dilemmas (e.g., Greene et al., 2001, 2004; Shenhav and Greene, 2014), the ventromedial prefrontal cortex (vmPFC) plays a central role. This replicable observation has led some (Moll and de Oliveira-Souza, 2007; Mendez, 2009) to suggest that the vmPFC is at the center of a prosocial neural network that codes for morally appropriate responses to harm-doing. For example, persons with lesions in the vmPFC are more open to pushing an innocent person to death in order to save others’ lives in the footbridge version of the trolley problem (Koenigs et al., 2007), a response that is otherwise seen as an unjustified form of violence. Based on such evidence, Moll and de Oliveira-Souza (2007) argue that vmPFC activation reflects an aversive emotional response to harmdoing that promotes prosocial behavior (namely not killing an innocent person). Greene (2007) also focuses on the vmPFC, but in that model, the region is posited to register conflict between harming an innocent person and utilitarian action to save more lives.

Despite the plausibility of Moll and de Oliveira-Souza’s interpretation, it is not altogether clear that the vmPFC has the explicit role of guiding prosocial behavior. One could argue that both moral norms against killing and utilitarian ethics are prosocial under the right circumstances. Thus, the viewer of violence will still have to decide whether the person engaging in violence is justified in doing so. It is here that another model of moral evaluation may be relevant. This model based on Aristotelian ethics (Casebeer, 2003) suggests a different role for the vmPFC. According to this model, the virtues reflected in characters’ motives determine the evaluation of their behavior. When characters exhibit noble motives, their behavior is seen as acceptable whether it is undertaken in support of moral norms or utilitarian purposes. Moll and de Oliveira-Souza’s interpretation of the pro-social function of the vmPFC is anti-utilitarian as suggested by results of lesion studies in the footbridge dilemma (Koenigs et al., 2007). However, from a virtue-ethics perspective, even if someone engages in violence for a utilitarian purpose, it may be seen as acceptable, such as in defense of self or others. In this approach, the vmPFC is more certainly to function as part of a neural system that responds to rewarding events whether they involve the self or others as this region is known to be involved in social decision making (Ruff and Fehr, 2014). Thus, rather than reflecting aversive emotion or conflict toward the killing of others, vmPFC can be seen as part of a network that responds to justifiable motives for harmdoing and that would be expected to track a film character’s engagement in violence if it furthers a social good, such as defense of self or others.

Based on a virtue-ethics approach, one should expect the vmPFC to respond to justified film violence; however, it is less clear what its role would be for unjustified violence. Research on brain responses to violent videos suggests that watching brief video clips of violence elicits activation of the lateral orbital frontal cortex (lOFC) (Kelly et al., 2007; Strenziok et al., 2010; Alia-Klein et al., 2014). Such brief portrayals of violence may well appear unjustified given that viewers are unlikely to have a context to judge its justification and may therefore assume that the violence is initiated by the violent character rather than being an act of self-defense. Consistent with this interpretation, activation in lOFC has also been observed when people imagine themselves attacking innocent persons in a video game (Molenberghs et al., 2016).

Given the oft-observed activation in lOFC in response to unjustified violence, we hypothesize that when a character engages in violence without a socially justified motive, attention focuses on the harm inflicted on the victims and vmPFC decreases in activity. At the same time, lOFC responds reflecting disapproval of the unjustified behavior. This response parallels the role of the vmPFC in that lOFC tends to react more strongly to aversive events (Berridge and Kringelbach, 2013; Rolls, 2015) while vmPFC responds more to reward. Thus, the motives of the characters engaged in movie violence may determine which region of ventral PFC is elicited.

In our study, we exposed participants to both justified and unjustified film violence so that we would be able to detect differences in vmPFC versus lOFC response to these different forms of violence. Our prediction is that justified violence will elicit significant response in vmPFC while unjustified violence will elicit significant response in lOFC. In order to test these predictions, we exposed late adolescents who frequently watch violent entertainment to examples of scenes from popular movies that involved either justified or unjustified violence. We chose this population in order to observe the effects of violent movies on viewers who are most likely not to be emotionally disturbed by exposure to violent content. At the same time, our research with adults (Romer et al., 2018) showed that repeated exposure to justified movie violence enhanced the acceptance of such violence relative to unjustified violence and therefore could maximize our ability to observe the same phenomenon in young people. The scenes were selected based on the criterion that justified violence was an acceptable response to prior aggression or wrongdoing (i.e., defense of self or others), and that unjustified violence had no apparently acceptable motive. These characteristics were verified by independent ratings (Romer et al., 2018). For each movie, we first showed the characters engaged in interaction prior to violence followed by a clip of the violent scene. Our reasoning motivating this experimental manipulation was twofold. First, we aimed to enable viewers to begin an evaluation of the motives of the characters and second, we aimed to enable a comparison of brain response between the scenes with and without violence that nevertheless involved the same characters.

In comparing the models, we used intersubject correlation (ISC) to identify the degree to which regional brain activity became synchronized across viewers in response to movie viewing (Hasson et al., 2004). ISC is a model-free approach to fMRI time series data that uncovers similarities of response across viewers without the need for comparisons with a control condition (Wilson et al., 2008). ISC is particularly suited to studying movies, which require sensitivity to neural activity over relatively long-time windows of narrative content rather than to short-term changes in sensory stimulation (Hasson et al., 2010; Pajula et al., 2012). The approach is also informative for exploring how differences in the violent content of popular movies are perceived by audiences. The approach has also been shown to be more sensitive to the narrative properties of a movie than to its surface features (Pajula et al., 2012; Nguyen et al., 2019) and to induce synchrony in brain regions that are sensitive to narrative content (Nummenmaa et al., 2012; Schmälzle et al., 2013). If the motives of the characters in violent movies determine brain responses as predicted by a virtue ethics approach, there should be differential synchronization of lOFC and vmPFC, with significant synchronization of vmPFC for justified violence and lOFC for unjustified violence.

## Materials and Methods

### Participants

Twenty-six late adolescents (mean age: 20.08 years, standard deviation: 1.08 years, 13 females) with an interest in watching violent movies were recruited to participate from two college campuses. All participants were native English speakers with at least a high-school degree and with normal vision and hearing. Participants were excluded for any history of neurological or psychiatric disorders, use of drugs or medications, and MRI contraindications. This study was reviewed and approved by the Institutional Review Board of the University of Pennsylvania. Participants provided written informed consent in accordance with the guidelines of the Institutional Review Board of the University of Pennsylvania (IRB No: 825895). Participants were compensated at a rate of $20 upon completion of the study.

### Experimental Setup and Procedure

A day before the MRI scan and after providing written informed consent, participants were asked to complete a set of computerized questionnaires that included demographic information, information relevant to participating in an MRI session, and a measure of callous affect, a facet of psychopathy (PS) taken from the Self Report Psychopathy scale (Paulhus et al., 2009). A second personality measure of various empathic tendencies (Davis and Davis, 1980) was assessed on the day of scanning. As stated in the recruitment flyer and explained in detail in the informed consent, participants were informed that they would be watching movies with violence, including scenes with gun violence.

We created two sets of movie segments, each containing four 90-s scenes involving gun violence preceded by a 90-s segment of the same characters only engaging in conversation. Along with rest and ratings, each movie condition was about 24 min long (see Supplementary Figure S1). The clips were taken from popular movies rated by the Motion Picture Association of America as either Parents Strongly Cautioned (PG-13) or Restricted (R) to children under the age of 17 unless accompanied by an adult. The clips were selected from a larger collection based on ratings of justification for the main character’s violence that we obtained from young adults using the Amazon Mechanical Turk online survey system. After viewing each clip, participants were shown a picture of the main character engaged in gun violence and asked: *Based on what you just saw, do you think the character pictured was justified in what they did?* Responses to this question were given on a 1 (not at all) to 5 (very much) scale. Movie clips that were rated high *versus* low on this scale were then tested with a national adult internet panel (*N* = 610) (Romer et al., 2018) that was randomly assigned to view either a set of four justified or four unjustified videos. These viewers verified that the two sets of videos differed in justification for violence, 3.82 ± 1.67 versus 2.67 ± 1.57, *F*(1,608) = 67.4, *p* < 0.001. Thus, the clips had clear examples of either justified or unjustified gun violence and were matched on victims of either sex. The clips were also matched as much as possible for sound and picture quality (see Supplementary Information and Supplementary Table S1; the movie clips are available at https://goo.gl/mc7hBt).

Gun violence occurs frequently in popular PG-13 movies and in contrast to R-rated movies, tends not to show the effects of the violence (e.g., blood and suffering). However, both justified and unjustified violence can occur in both types of movies. We removed signs of blood and suffering from the R-rated films to make them comparable to the PG-13-rated films so that the narrative characteristics of the characters rather than the consequences of the violence were the primary difference between the justified and unjustified conditions.

The full experimental design is shown in Supplementary Figure S1. The participants were asked to relax in the MRI machine for 5 min before viewing either four sets of justified (J1) or unjustified (U1) clips, each composed of a 90-s segment of the main characters without gun violence (character) followed by a 90-s segment of the characters engaged in violence (action) in the same movie (see Supplementary Figure S1). Displaying the character segment before the action segment gave participants additional exposure to the narrative properties of the characters before they engaged in violence (see Supplementary Table S1). Participants were randomly assigned to view one set of clips first (either J1 or U1), followed by the other set (either U2 or J2). As shown in Supplementary Figure S1B, there was a 5-s interlude between segments to prevent any overlap in the blood oxygenation level-dependent (BOLD) signal, and during this interlude text was displayed to inform participants of the next video’s content (either character or action). The order of the clips was randomly assigned for each participant, and the order of the clips within each set was randomly determined with the same clips in J1 and J2, and likewise in U1 and U2. The experiment lasted for approximately 1 h in the scanner.

During scanning, stimulus presentation was controlled by a computer with Neurobehavioral Systems (NBS) software^1^. Participants were given a set of headphones. The decibel level transmitted through the headphones was calibrated for each participant to ensure that they were able to hear the audio. The movie clips and instructions were displayed on a screen projected from the rear of the scanner. During the resting periods, a blank screen was displayed. After the set of four clips in each condition was shown, participants were shown a still photo of the main characters in each movie shooting a weapon and were asked how the character made them feel using a scale going from 1 (very bad) to 4 (good). The participants were provided with the keypad to respond to appropriate answer. This assessment was designed to verify that the characters in the justified condition would be seen in a more favorable light despite their use of force.

### fMRI Data Acquisition

Magnetic resonance images were obtained at the Hospital of the University of Pennsylvania (HUP) using a 3.0 T Siemens Trio MRI scanner equipped with a 32-channel head coil. T1-weighted structural images of the whole brain were acquired on the first scan session using a three-dimensional magnetization-prepared rapid acquisition gradient echo pulse sequence [repetition time (TR) 1810 ms; echo time (TE) 3.50 ms; voxel size 1 mm × 1 mm × 1 mm; matrix size 192 × 256 × 160]. This first scan represents the anatomical reference. In all experimental runs while watching video clips, T2^∗^-weighted images sensitive to BOLD contrasts were acquired using a slice accelerated multiband echo planar pulse sequence (TR 2000 ms; TE 25.2 ms; flip angle 60°; voxel size 2 mm × 2 mm × 2 mm; field of view 208 mm; matrix size 104 × 104 × 80). In all resting state runs, T2^∗^-weighted images sensitive to BOLD contrasts were acquired using a slice accelerated multiband echo planar pulse sequence (TR 500 ms; TE 30 ms; flip angle 30°; voxel size 3.0 mm × 3.0 mm × 3.0 mm; field of view 192 mm; matrix size 64 × 64 × 48).

### fMRI Data Preprocessing

Cortical reconstruction and volumetric segmentation of the structural data was performed with the Freesurfer image analysis software (Dale et al., 1999). Boundary-Based Registration between structural and mean functional image was performed with *bbregister* in Freesurfer (Greve and Fischl, 2009). Preprocessing of the fMRI data was carried out using FEAT (FMRI Expert Analysis Tool) Version 6.00, part of FSL (FMRIB’s Software Library). The following preprocessing steps were applied: motion correction using MCFLIRT (Jenkinson et al., 2002); slice-timing correction using Fourier-space time series phase-shifting; non-brain removal using BET (Smith, 2002); grand-mean intensity normalization of the entire 4D dataset by a single multiplicative factor; high-pass temporal filtering (Gaussian-weighted least-squares straight line fitting, with sigma = 60.0 s). Nuisance time series were voxel-wise regressed from the preprocessed data. Nuisance regressors included (i) three translation (*X*, *Y*, *Z*) and three rotation (α, β, γ) time series derived by retrospective head motion correction, together with expansion terms, for a total of 24 motion regressors (Friston et al., 1996); (ii) the first five principal components of non-neural sources of noise, estimated by averaging signals within white matter and cerebrospinal fluid masks, obtained with Freesurfer segmentation tools and removed using the anatomical CompCor method (aCompCor) (Behzadi et al., 2007); (iii) local noise estimated by averaging signals derived from the white matter region located within a 15 mm radius from each voxel, using the ANATICOR method (Jo et al., 2010). In addition, we applied spatial smoothing with a Gaussian isotropic kernel of 5-mm full width half maximum.

### Time Series Data

Time series were extracted for each participant from a finer grained template of 626 anatomical regions of interest (ROIs) (Hermundstad et al., 2013) defined by an upsampled version of the 116-region AAL atlas (Tzourio-Mazoyer et al., 2002) covering the whole brain including cortical, subcortical, and cerebellar regions as well as vermis. This 626 AAL anatomical atlas has been used in previous studies for both resting state and task investigations (Hermundstad et al., 2014; Bassett et al., 2015). The 626 AAL atlas in Montreal Neurological Institute (MNI) space was registered to the each subject functional data through anatomical image before the extraction of the regional time series to preserve individual differences. The regional mean times series were filtered between 0.05 and 0.1 Hz for further analysis.

### Intersubject Correlation Analysis and Statistical Analysis

**FIGURE 1:**
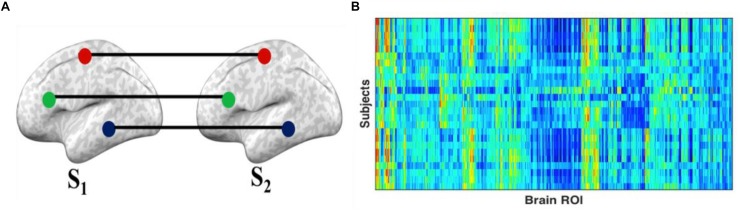
Intersubject correlation (ISC): **(A)** ISC is the correlation of the activity of the same region of interest across all subjects. The ISC is estimated by the correlation of each subject’s (S_1_) ROI time series and the average ROI time series of all other subjects (S_2_ … S_*N*_). **(B)** The final results can be represented in a matrix with rows representing subjects and columns representing ROIs, with the cells containing the ISC of each ROI for each subject.

Intersubject correlation (Hasson et al., 2004) is a model-free approach to measure synchrony of stimulus-driven response across subjects. Generally, ISC estimates the correlation between the same voxels or region in a times series of hemodynamic activity between two subjects (Figure 1). The ISC of a particular ROI was calculated using the Pearson correlation of the time series of the first subject (S1) and the average time series of all other subjects, S_*N(*__∉__*S*__1__)_ (Simony et al., 2016). This operation is repeated for all brain ROIs and for all subjects while watching movie clips and during resting conditions. The procedure measures the extent of similarity of functional activity of each subject to all other subjects.

The statistical significance of each correlation between two ROIs was assessed by a permutation procedure based on surrogate data. The surrogate data were generated by phase-randomizing BOLD time series while maintaining the mean and autocorrelation of the original signal. The null distributions of correlation values were obtained via comparison to 10000 surrogate BOLD time series. The family wise error rates (FWER) were controlled by defining a threshold at the *q* × 100th percentile of the null distribution of maximum values. The thresholds for each condition were at *q* = 0.001.

We calculated ISC across the participants to investigate brain synchrony in BOLD responses while watching both justified and unjustified movie violence. Because the action segments could potentially elicit different inter-subject synchrony of BOLD responses in comparison to character segments, we computed the ISC for action and character segments separately. Differences between action and character segments were determined by stringent statistical testing to determine the effect of violence on intersubject synchronization.

Following our hypothesis that the portrayal of violence as justified or unjustified would elicit different neural patterns of ISC, we compared the ISC of justified movie violence with that of unjustified movie violence. We also examined differences between action and character segments within the same movies to ensure that the differences between conditions were attributable primarily to the violent segments rather than to the characters that were engaged in the action, and we examined ISC for each of the four movie clips separately to verify the stability of differences by condition.

The significance of the ISC across and between subjects was estimated by a *t*-test with an FWER multiple comparison correction applied across 10000 randomizations. All statistical tests reported are two-tailed unless otherwise stated.

The ISC was computed with custom MATLAB scripts and statistics analyses were done using R 3.5.1.

## Results

### Character and Other Ratings

A questionnaire administered before the experiment verified that all participants regularly watch movies with violence with an average of 2.5 h per day. In addition, 70% indicated that they play active shooter video games. After the experiment, participants were asked if they could remember having seen any of the movies that were shown in the scanner. Surprisingly, only one movie that featured *James Bond* was remembered by all participants and more than 80% couldn’t remember if they had seen any of the other clips even with more description from the research coordinators. Consistent with our selection of the clips, a paired-samples *t*-test revealed that participants reported feeling worse (*t*(24) = 3.84, *p* = 0.00078) about the major characters using a weapon in the unjustified condition (*M* = 2.27, SD = 0.37) than in the justified condition (M = 2.67, SD = 0.38).

### Intersubject Correlation and Neural Patterns While Watching Movie Violence

**FIGURE 2:**
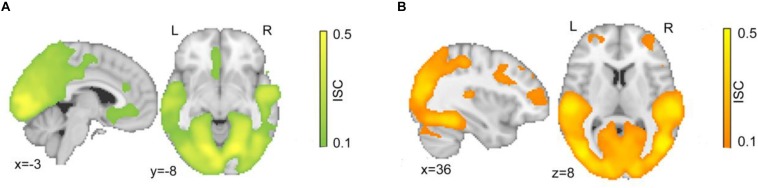
Intersubject correlation for both character and action segments. Significant ISC maps for **(A)** justified and **(B)** unjustified movie violence for each 30-min period. Both conditions showed significant ISCs in occipital and temporal cortex associated with visual and auditory processing. The justified condition specifically elicited significant ISC in vmPFC, while the unjustified condition specifically elicited significant ISC in frontal regions including lOFC. The color bar provides the magnitude of ISC values, with a significance threshold set at *r* < 0.1, after applying a non-parametric family wise error, FWE, correction of *q* < 0.001.

Participants watched a series of clips depicting either justified or unjustified movie violence. In the first step, the overall ISC of hemodynamic activity of the set of 26 subjects was calculated during viewing of both the character and action segments of justified (Figure 2A) and unjustified movie violence (Figure 2B). In both conditions, significant ISCs were observed in occipital lobes, posterior parietal areas, and temporal cortices; these regions are well known to be synchronized across participants while watching movies (Hasson et al., 2004). The justified condition (Figure 2A) revealed significant ISC at the vmPFC, as well as anterior cingulate cortex (ACC). The unjustified condition (Figure 2B) elicited significant ISC in lOFC, as well as inferior frontal gyrus (IFG), superior frontal gyrus (SFG), and medial frontal gyrus (MFG).

Table 1 provides further details about the regions characterized by ISC values that were significantly greater or lesser than the null, as assessed by cluster analysis; variables reported include the hemisphere of the cluster, the cluster size, and the ISC values of the voxel with the highest value within the cluster. Significant ISCs covered both primary and secondary visual and temporal regions for both justified and unjustified movie violence. Other regions that displayed significant ISC included the SFG and the middle fontal gyrus. However, as shown in Figure 2A, the vmPFC’s ISC was significant only in the condition of justified movie violence and the ISC of lOFC was only significant for unjustified violence.

**TABLE 1:** Justified versus unjustified violence.

**Anatomical regions**	**Hemisphere**	**Voxels**	**ISC**	**MNI coordinates (mm)**		
				
				**X**	**Y**	**Z**
**Justified**						
Occipital and temporal cortex	R/L	60968	0.591	42	−64	−4
Superior frontal gyrus	R	771	0.157	12	16	46
Ventral medial prefrontal cortex	L	751	0.154	−14	8	−20
Precentral cortex	L	713	0.193	−40	−6	32
Frontal pole	L	515	0.181	−32	62	−2
Middle frontal gyrus	R	345	0.168	50	4	20
Inferior frontal gyrus	R	258	0.166	52	22	10
Inferior temporal gyrus	R	205	0.151	44	−10	−46
Superior frontal gyrus	L	200	0.247	−24	−6	46
**Unjustified**						
Occipital and temporal cortex	R/L	49105	0.507	−42	−86	−8
Middle frontal gyrus	R	641	0.186	34	0	36
Anterior cingulate cortex	R/L	369	0.163	0	32	12
Frontal pole	R	341	0.153	32	42	20
Lateral orbital frontal cortex	R	318	0.157	38	38	6
Posterior cingulate cortex	L	234	0.162	−6	−32	32
Precentral cortex	L	223	0.157	−40	−6	32
Superior frontal gyrus	L	200	0.187	−24	−6	46

*The table lists the anatomical regions, hemisphere, number of voxels, corresponding ISC values of the voxel with highest value within the cluster, and corresponding peak voxel coordinates in MNI space.*

### Justified Versus Unjustified Violence During Action and Character Segments

**FIGURE 3:**
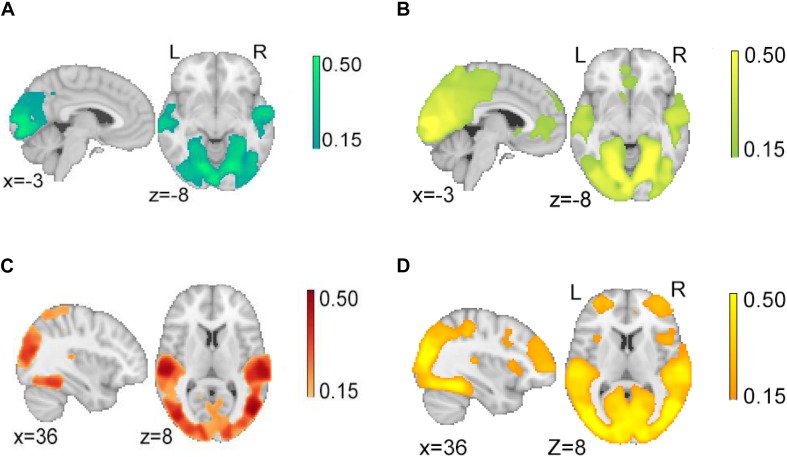
Intersubject correlation (ISC) in character and action segments of justified and unjustified movie violence. We observed significant ISCs in temporal and occipital cortex associated with audio and visual processing for both **(A)** character and **(B)** action segments of justified movie violence. In contrast, we observed significant ISC in vmPFC and ACC of **(B)** action segments only. Similarly, we observed significant ISCs in temporal and occipital cortices related to auditory and visual processing for both **(C)** character and **(D)** action segments of unjustified movie violence. In contrast, we observed significant ISC in lOFC, MFG, and insula regions of **(D)** action segment only. The color bar provides ISC values, with a significance threshold set at *r* < 0.15, after applying a non-parametric family wise error, FWE, correction of *q* < 0.001.

In the second step, we investigated the ISC observed while participants watched the character and action segments of justified and unjustified movie violence (Figure 3). In both character (Figure 3A) and action (Figure 3B) segments of justified movie violence, significant ISC was observed in broad swaths of temporal and occipital cortices associated with visual and auditory processing. In contrast, we observed significant ISC in vmPFC and ACC for action segments only (Figure 3B). Similarly, in the unjustified condition, we observed significant ISC in broad swaths of temporal and occipital cortices associated with visual and auditory processing, for both the character (Figure 3C) and action (Figure 3D) conditions. Notably, significant ISC was observed in lOFC, MFG, SFG, and bilateral insula regions during action segments only (Figure 3D). For both justified and unjustified movie violence, the global ISC during action segments was significantly higher (*p* < 0.0001) compared to the global ISC during character segments (Supplementary Figure S2).

Cluster analyses of ISC of character and action segments (Table 2) revealed that the ISC of primary and secondary occipital and temporal regions of both were significant for both movie conditions. ISCs of frontal and subcortical regions were only significant during action segments across the subjects. In addition to occipital and temporal regions associated with auditory and visual processing, ISC was significant in vmPFC, ACC, hippocampus, and bilateral caudate regions for action segments of justified movie violence. The unjustified movie violence revealed significant ISCs in bilateral lOFC and insula, and ACC for action segments.

**TABLE 2:** Character versus action.

**Anatomical regions**	**Hemisphere**	**Voxels**	**ISC**	**MNI coordinates**		
				
				***X***	***Y***	***Z***
**Justified character**						
Occipital and temporal cortex	R/L	23777	0.57	42	−64	−4
Superior temporal gyrus	L	3034	0.422	−46	−20	−8
Precuneus	R	1252	0.268	2	−54	48
**Justified action**						
Occipital and temporal cortex	R/L	64912	0.602	−12	−78	−14
VMPFC/Anterior cingulate cortex	R	2533	0.257	14	50	16
Superior frontal gyrus	R	1069	0.271	12	16	46
Middle frontal gyrus	R	634	0.254	50	4	20
Inferior frontal gyrus	R	270	0.252	62	22	−2
Hippocampus	L	249	0.203	−30	−6	−28
Superior frontal gyrus	L	200	0.362	−24	−6	46
Caudate	R	188	0.212	14	0	12
Caudate	L	181	0.211	−10	−4	14
**Unjustified character**						
Occipital and temporal cortex	R/L	22901	0.492	52	−76	0
Superior temporal gyrus	L	2907	0.473	−50	−28	2
Lateral occipital cortex	R	1096	0.253	26	−56	48
Supramarginal (TPJ)	R	516	0.203	46	−38	28
**Unjustified action**						
Occipital and temporal cortex	R/L	64821	0.632	−42	−86	−8
Lateral orbital frontal	R	3099	0.327	36	46	−2
Lateral orbital frontal	L	2739	0.336	−34	42	−2
Anterior cingulate cortex	R/L	1429	0.264	0	32	12
Insula	L	1328	0.25	−36	20	8
Insula	R	849	0.262	52	12	−2
Superior frontal gyrus	R	246	0.205	12	30	38
Precentral	L	223	0.223	−40	−6	32

*The table lists the anatomical regions, hemisphere, number of voxels, corresponding ISC values of the voxel with highest value within the cluster, and corresponding peak voxel coordinates in MNI space.*

### The Neural Pattern of ISC During Character and Action Segments of Movie Violence

In the third step, we assessed the robustness of ISC results by examining ISC patterns of character and action segments for each clip in each of the justified and unjustified conditions. This was done to investigate whether a particular movie (irrespective of viewing positions or order) influenced significant ISC especially during action segments. It was our prediction that ISC would be associated with the distinctive narratives of justified and unjustified actions.

**FIGURE 4:**
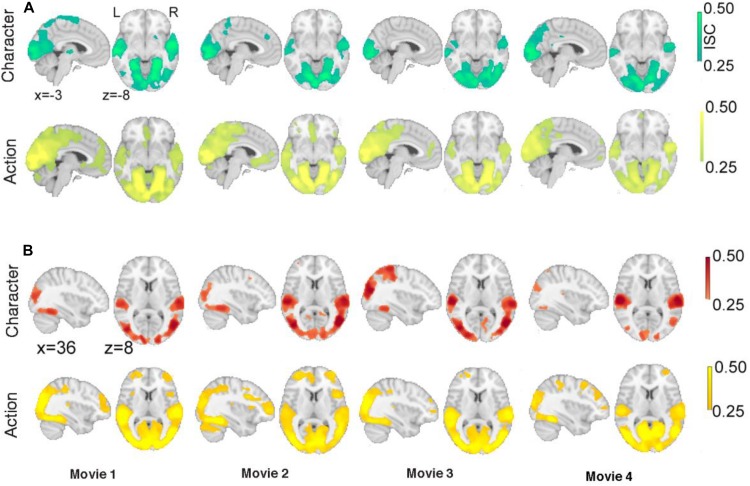
Intersubject correlations during action segments compared to during character segments. Significant ISCs of **(A)** justified movie clips for character and action segments and **(B)** unjustified movie clips for character and action segments. The color bar provides ISC values, with a significance threshold set at *r* < 0.25, after applying a non-parametric family wise error, FWE, correction of *q* < 0.001.

Figure 4 shows the significant ISC for both justified and unjustified movie clips. For justified movie conditions (Figure 4A), both character and action revealed significant ISC in occipital and temporal cortices associated with visual and auditory processing. In contrast, we observed significant ISCs in frontal regions especially at vmPFC and ACC during action segments only. Moreover, for unjustified movie violence (Figure 4B), we observed significant ISC in occipital and temporal cortices associated with visual and auditory processing for both character and action segments. Meanwhile, during action segments of unjustified movie violence, significant ISC was observed in lOFC, SGF, MFG, and insula.

Collectively, these results demonstrate that ISCs were stronger in frontal regions during action segments but were different for justified and unjustified movie violence. Such evidence supports the notion that the ISC of hemodynamic signals reflects subjects’ brain responses that differentiate justified versus unjustified violent actions in the movies.

### Differences Between ISCs in lOFC Versus vmPFC

**FIGURE 5:**
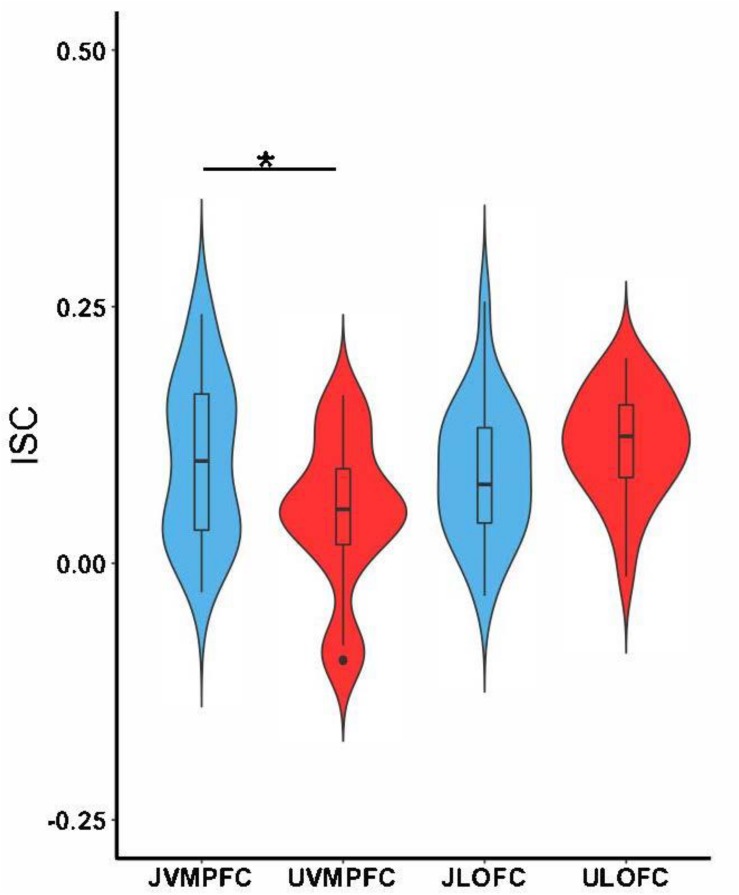
Tests of differences collapsing over character and action segments. The boxplots show the mean and the standard deviation. The ISC in the vmPFC was significantly higher while participants watched justified movie violence (JvmPFC) compared to when participants watched unjustified movie violence (UvmPFC). There was no significant difference between ISC in lOFC for justified (JlOFC) versus unjustified (UlOFC) movie violence (^∗^*p* < 0.05).

We hypothesized that viewing justified and unjustified movie violence would reveal significant ISC at vmPFC and lOFC, respectively. Figures 2–4 support these predictions, particularly during action segments. We tested these predictions more directly by first examining the values of ISC across character and action segments for both conditions (Figure 5). The ISC in vmPFC was significantly higher (*p* = 0.017) in the justified movie condition (JvmPFC) compared to the unjustified movie condition (UvmPFC). Although the lOFC ISC was higher for unjustified (UlOFC) compared to justified movie violence (JlOFC), it did not reach statistical significance (*p* = 0.12). However, the two-way ANOVA (character versus action and justified versus unjustified) revealed a significant pattern of regional (vmPFC and lOFC) synchronization across the groups (*F*_2_,_9__6_ = 6.483, *p* = 0.0024) but no significant effect between the groups (*F*_1_,_96_ = 0.98, *p* = 0.324) for both vmPFC and lOFC during both character and action.

**FIGURE 6:**
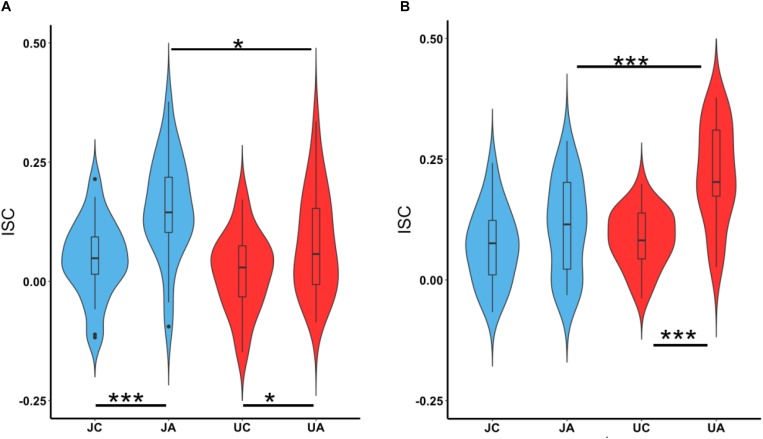
vmPFC and lOFC ISC during character and action segments. The boxplots show the mean and the standard deviation. **(A)** The ISC in vmPFC for character and action segments for both justified and unjustified movie violence. **(B)** The ISC in lOFC for character and action segments for both justified and unjustified movie violence. (JC, justified character, JA, justified action, UC, unjustified character, UA, unjustified action, ^∗^*p* < 0.05, ^∗∗∗^*p* < 0.001).

We also investigated the ISC of vmPFC and lOFC while participants watched the character and action segments separately. Figure 6A shows the ISC in vmPFC while watching both justified and unjustified movie violence. A two-way ANOVA (character versus action and justified versus unjustified) revealed significant differences between character and action (*F*_2_,_96_ = 10.40, *p* = 8.18 × 10^–5^) as well as between justified and unjustified violence (*F*_1_,_96_ = 7.14, *p* = 8.85 × 10^–3^). The vmPFC ISCs for action segments (JA, UA) were significantly higher than their corresponding character (JC, UC) segments (JA > JC: *p* = 0.0002 and UA > UC: *p* = 0.027). Interestingly, the vmPFC ISC was significantly higher for justified action (JA) segments compared to unjustified action (UA) segments (JA > UA; *p* = 0.019).

For lOFC (Figure 6B), the two-way ANOVA also revealed significant differences between character and action (*F*_2_,_96_ = 16.75, *p* = 5.734 × 10^–7^) as well as between justified and unjustified violence (*F*_1_,_96_ = 12.30, *p* = 4.97 × 10^–4^). The ISC was significantly higher during action segments compared to character segments of unjustified violence clips (lOFC: UA > UC; *p* = 0.000001). We observed no difference between the character and action segments for justified violence. Interestingly, the ISC in lOFC for action segments was significantly higher for unjustified violence compared to justified violence (UA > JA; *p* = 0.0003).

## Discussion

We found brain synchronization across participants when viewing dynamic scenes of violence in movies, with results that were replicable across different movies. We were particularly interested to observe differences in synchrony for vmPFC and lOFC, each of which has been linked to differential approval of morally relevant behavior. Consistent with a virtue-ethics model, we found differential synchrony, such that justified violence elicited significantly greater ISC in vmPFC than unjustified violence, whereas unjustified violence produced greater ISC in lOFC than justified violence. Consistent with this interpretation, participants reported feeling better about the characters shooting their weapon in the justified than unjustified condition.

The finding that unjustified violence elicited significantly greater ISC in lOFC is consistent with considerable research showing activation in this region in response to brief video segments depicting violence (Kelly et al., 2007; Strenziok et al., 2011; Alia-Klein et al., 2014). These brief clips were likely seen as unjustified because they did not permit the attribution of virtuous motives to the actors. The finding is also consistent with Molenberghs et al. (2016) in which video game players imagined engaging in unjustified shooting of civilians. According to a virtue-ethics model, there was no significant synchronization across viewers in vmPFC for unjustified violence because the motives of the harm-doers exhibited little in the way of acceptable reasons for their behavior. Under this condition, synchrony of lOFC was the more dominant response, likely reflecting collective aversion to their behavior. This response is consistent with the finding that activation of lOFC has been associated with responses to relatively aversive events (Berridge and Kringelbach, 2013; Rolls, 2015), including violations of social norms (Berthoz et al., 2002).

The synchronization of vmPFC in response to justified movie violence indicates that the vmPFC is especially sensitive to characters who are seen as justified in using violence, as predicted by a virtue ethics approach. Our findings are less consistent with the approach advocated by Moll and de Oliveira-Souza (2007) and Mendez (2009) who regard this region as a center for prosocial moral evaluation. In their model, the vmPFC registers aversion to violation of norms against killing; whereas, the virtue-ethics approach regards the vmPFC as associated with more general approval of behavior that in this case reflects the virtuous motives of movie characters. Activation in this region is associated with response to rewarding events, whether regarding oneself or others (Ruff and Fehr, 2014), a response that is likely to be elicited by many types of behavior, including those motivated by virtuous motives.

The virtue-ethics approach is also differentiated from the model proposed by Greene (2007), which also regards the vmPFC as opposed to utilitarian action. This response is said to arouse conflict with fronto-parietal circuits that support the utilitarian choice. However, we saw no evidence of such conflict when violence was justified, perhaps because the utilitarian consequences (stopping a harm-doer) were not in conflict with acting in self-defense. In addition, Greene’s model would expect synchrony in the vmPFC in the unjustified condition because it involved a violation of the moral norm against hurting innocent persons. In total, we found more evidence in support of the virtue model, which focuses on the motives of the harm-doer, than in support of Greene’s model, which posits conflict between vmPFC and fronto-parietal regions.

The more recent model of Shenhav and Greene (2014) proposes that the vmPFC integrates input from the amygdala, which is inversely related to utilitarian action, and systems that support utilitarian action. Our results indicated that the vmPFC exhibited synchrony when violence was justified. However, their model would seem to predict greater synchrony in vmPFC and amygdala when violence was unjustified; nevertheless, we mainly observed synchrony in the lOFC and regions that are activated when empathically experiencing the pain of others, namely insula (Singer et al., 2004, 2006), suggesting that participants empathized with the pain of the victims of unjustified violence. Insula is recognized as a region that responds to pain, disgust, and mood in other persons (Singer et al., 2004; Decety and Porges, 2011), especially the anterior insula, a region that encodes bodily reactions to events (Gu et al., 2012). This reaction would be less expected in the justified condition because the victims of justified violence would be seen as more deserving of retaliation.

### Global Differences in Synchrony

Across the whole brain, we observed that brain synchrony was greater while watching action segments in comparison to character segments for both justified and unjustified violent movies. Regionally, the neural pattern showed that action segments elicited greater cross-subject synchronization in both primary and secondary visual cortices, including areas implicated in visual-spatial processing and visual memory encoding. This finding is not surprising in view of the entertainment industry’s heavy emphasis on violent programing and its success in drawing viewers (Hamilton, 2000). It is intuitively plausible that narrative features associated with violence, such as strong visuals and emotionally arousing action, have the ability to elicit wide-spread synchronization across viewers (Bezdek et al., 2015). It is also consistent with Nummenmaa et al. (2012), who found greater ISC associated with emotional arousal but not emotional valence. Action segments would be expected to be more arousing than character segments irrespective of the justification of violence; but synchronization would not be expected in the same regions in response to justified versus unjustified violence, which differ considerably in the valence of the behavior.

### Implications for Imitation of Violence

Our results suggest that late adolescents who are accustomed to viewing violent entertainment also exhibit brain synchronization reflective of acceptance of violence when it is seen as justified. The finding that brain synchrony discriminated between justified and unjustified violence suggests that even youth who are attracted to such content are sensitive to its moral implications. It remains for future research to determine whether the brain responses to justified film violence we have observed foster tendencies to imitate or consider the use of weapons for self-defense or other justified purposes. Laboratory research finds that justified film violence can encourage aggressive responses in response to provocation (Berkowitz, 1984). What is less clear is whether the use of guns in movie portrayals of justified violence encourages their acquisition and use for purposes of self-defense.

It is noteworthy that we also observed heightened synchrony in vmPFC during unjustified violence compared to the character segments. This synchrony was weaker than for justified violence and may suggest some level of favorable reaction to this kind of violence in this sample of heavy viewers of media violence. Future research should explore this possibility by comparing heavy versus lighter users of violent media. It is possible that heavy use of violent media leads to greater acceptance of even unjustified violence.

### Limitations and Future Directions

We focused on popular movies with violence that is considered acceptable for wide audiences. Future research could examine movies in which the violence is more graphic to determine whether this affects synchrony in OFC and other regions. It is possible that such violence evokes less synchrony in vmPFC even if it is justified, although from a virtue perspective, this should matter less than the motives and intentions of the characters engaging in the violence. These questions require more attention in future studies. In addition, our use of actual movie clips reduced our ability to control other aspects in comparisons between justified and unjustified violence, such as the scenes, actors, and gun shootings. However, the use of ISC should minimize these concerns. This method is more sensitive to narrative characteristics of stimuli, such as found in movies than to the details of scenes and actors that do not affect the narrative flow of the action (Hasson et al., 2010; Nguyen et al., 2019). For example, it should matter less when the violence in a scene occurs than the recognition of the motives of the character in the narrative. Indeed, we found the same patterns across a wide range of movies and orders of viewing, suggesting that these factors were not responsible for the findings.

In addition, our video clips were segments of the whole movie. Cutting and editing movie segments can alter ISC especially in the primary visual and auditory regions (Herbec et al., 2015). This may result in higher ISC observed during action segments especially in the visual region. The higher ISC during action segments can also be due to the frequent appearance of high interest objects such as guns and rapid movement associated with fighting.

It is also important to note that we did not study peaks in activation of brain response but rather similar time-series responses to the narrative properties of movies. These model-free synchronies are of great interest in understanding the effects of different forms of narrative, which might not be evident in simply observing peaks in activation (Hasson et al., 2010). For example, comparing peaks of activation in movie scenes of violence to resting states may obscure the role of the vmPFC, which is part of the default mode network (Wilson et al., 2008). Using the ISC model-free approach removes this difficulty and allows a focus on changes within a narrative rather than between a narrative and a comparison of doubtful relevance. At the same time, comparing different narratives (justified versus unjustified violence) using ISC revealed interesting differences that can be used to test theories of moral evaluation.

Finally, although we focused on scenes with gun violence, the findings may not be unique to the use of guns, as other weapons are likely to produce similar results. Guns were a particular focus because of their widespread availability in the United States, a country with disproportionate injury resulting from their use, especially among young people (Grinshteyn and Hemenway, 2016).

## Conclusion

Our research is the first to demonstrate that when movie characters engage in violence seen as justified, there is significant synchronization in vmPFC, providing evidence in favor of the theory that the participants viewed the violent behavior as acceptable for self or family protection. However, significant synchronization of lOFC and insula regions across the participants while watching unjustified movie violence provided evidence in favor of the theory that participants rejected such acts of violent behavior. The findings indicate two unique patterns of neural synchrony while viewing violence that have not been the focus of prior research. Future research should continue to test predictions of a virtue-ethics approach to moral evaluation.

## Data Availability Statement

The datasets generated for this study are available on request to the corresponding author.

## Ethics Statement

Participants were excluded for any history of neurological or psychiatric disorders, use of drugs or medications, and MRI contraindications. Participants provided written informed consent in accordance with the guidelines of the Institutional Review Board of the University of Pennsylvania (IRB No. 825895).

## Author Contributions

AA, DB, PJ, and DR designed and conceived the experiments. AA and DB developed the analytical methods. AA performed the computations. DB and DR supervised the project. AA, DB, and DR wrote the manuscript in consultation with PJ. All authors discussed the results and contributed to the final manuscript.

## Conflict of Interest

The authors declare that the research was conducted in the absence of any commercial or financial relationships that could be construed as a potential conflict of interest.

## Footnotes

1 www.neurobs.com
