# Supplementary material for: Intersubject Synchronization of Late Adolescent Brain Responses to Violent Movies: A Virtue-Ethics Approach
Source: Front Behav Neurosci. 2019 Nov 22;13:260. doi: 10.3389/fnbeh.2019.00260 (PMC6885594; doi:10.3389/fnbeh.2019.00260)
Supplement: Supplementary file 1 [file Table_1.DOCX]

**SUPPLEMENTARY INFORMATION**

**Video clips and experimental setup**

The justified clips used for this study were *Die Hard, White House Down, Terminator Salvation,* and *Taken.* For unjustified clips*, Jack Reacher, Sky Fall, Sicario, and Training Day* were selected. The video clips are available at https://goo.gl/mc7hBt. The full experimental design is as shown in Figure S1. The participants were asked to relax in the MRI machine for 5 mins before viewing either 4 sets of justified (J1) or unjustified (U1) clips, each composed of a 90-sec clip of the main characters without gun violence (character) followed by a 90-sec clip of the characters engaged in violence (action) (see Figure S1a). The total time to watch all the clips took about 1 hour. Participants were randomly assigned to view one or the other set first followed by the other set (either J2 or U2). The J1 and J2 consisted of the same set of justified clips; likewise, U1 and U2 consisted of the same set of unjustified clips. All participants watched both justified and unjustified movies separated by 10 minutes rest in between each set of video clips. There was a 5-sec interlude between clips to prevent overlap of BOLD signal during which text was displayed to inform participants of the next video’s content (either character or action). As shown in Figure S1B, the character (C) segments were shown first followed by the action (A) segments. Displaying the character segment before the action segment of the same movie enabled analysis of reactions to the characters prior to their engaging in violence.

**Supplementary Figure 1. Video clips and experimental procedure** (a) For each of the four clips, 90 secs of character and action segments are displayed. The character segments consisted of a discussion between the actors subsequently shown engaging in violence and the action segments consisted of violent scenes involving use of guns and shooting. The character segments familiarized participants with the actors. (c) For each condition (justified or unjustified), participants watched eight 90-second video clips consisting of 4 pairs of character (C) and action (A) segments from the same movie. The participants were randomly assigned to two equal groups- the first group watched J1 first follow by U2 and the second group watched U1 first follow by J2. J1 and J2 consisted of the same set of clips; likewise, for U1 and U2.

**Supplementary Table 1. Summary of movies used to extract both justified and unjustified clips.**

| **Movie** | **Character** | **Action** |
| --- | --- | --- |
| **Justified violent movies** | | |
| Die hard (2007) | John McClane (Bruce Wills) is attempting to stop cyber terrorists who have hacked into government computers, but his daughter is kidnapped by the terrorists in attempt to stop him. | McClane confronts the terrorists who have his daughter. She tries to escape by shooting one of them and giving her father the gun. The lead terrorist takes the gun and threatens to kill McClane and his daughter, but McClane manages to shoot the terrorist instead. |
| White house down (2013) | The President of the US (Jamie Fox) is being attacked in the white house by armed terrorists. John Cale (Channing Tatum) is a police officer who tries to protect the President. | The two characters have to fight and kill the terrorists under the lockdown in the White House. The action involves heavy use of automatic weapons to stop the invaders. |
| Terminator salvation (2009) | Connor (Christian Bale) talks with his daughter about how to strike back at a catastrophic attack of robotic enemies. | In a bleak scene with a backdrop of destroyed battle equipment, Connor is attacked by a robot. He is almost captured but manages to command a powerful machine gun that he shoots to destroy the robot. |
| Taken (2008) | Bryan Mills (Liam Neeson) talks with his young daughter and wife prior to the daughter’s trip to Paris for a vacation. He expresses worry about her safety. | Mills is seen entering a heavily guarded hallway where he must first overcome the attacks of a guard before he breaks into a room where his daughter is being held by a criminal. Mills shoots the criminal, saving his daughter. |
| **Unjustified violent movies** | | |
| Jack Reacher (2012) | An ex-military sniper (Jai Courtney) is working for a mobster and is shown discussing his plans with a member of the mobster’s team. | The sniper is shown randomly killing ordinary people from a safe distance using a military grade rifle. His intent is to only kill the adversary but to cover up the motive for the crime, he shoots five people dead in a seemingly random attack. |
| Skyfall (2012) | James Bond (Daniel Craig) and a female accomplice are captured by the master-criminal Silva (Javier Bardem) who is seen menacingly interrogating him. | Silva places a glass on the accomplice’s head and threatens to shoot Bond if he does not shoot the accomplice. After much tension, Silva shoots the accomplice and kills her. |
| Sicario (2015) | FBI agent Kate (Emily Blunt) gets the background on a task force for the escalating war against drugs, led by the shadowy Alejandro (Benicio Del Toro). | Alejandro wearing a disguise interrupts a drug deal by shooting one of the dealers and taking another hostage. Kate appears trying to arrest both men but Alejandro shoots her repeatedly. Wearing a bullet-proof vest, she survives. |
| Training day (2001) | A veteran police officer (Denzel Washington) escorts a rookie (Ethan Hawke) on his first day on a drug bust. | The veteran orders the rookie to kill the dealer and when he refuses, the veteran does so himself. |

**Behavioral and self-assessment reports**

**Supplementary Table 2** shows the means of personality scores we obtained from each participant and the internal consistency for each score as revealed by Cronbach’s alpha. We investigated whether there was a difference in any of the measures between the two groups that had watched justified and unjustified clips in different orders and found no statistically significant differences between them. This suggested that random assignment to viewing order was successful.

**Supplementary Table 2.** Summary of self-assessment behavioral measures.

|  | **Behavioral Measures** | **Mean (Std)** | **Cronbach’s alpha** | **Statistical difference of order of presentation**  **(*t(12)*, *p*-value)** |
| --- | --- | --- | --- | --- |
| 1 | Psychopathy (PS) | 2.68 (0.45) | 0.9092 | 0.1264, 0.9017 |
| 2 | Fantasy Seeking (FS) | 3.40 (0.52) | 0.7131 | 1.0753, 0.3052 |
| 3 | Empathic Concern (EC) | 3.67 (0.69) | 0.8785 | 1.6325, 0.1285 |
| 4 | Perspective Taking (PT) | 3.53 (0.54) | 0.7677 | 1.6167, 0.1342 |
| 5 | Personal Distress (PD) | 2.88 (0.73) | 0.8464 | 0.3602, 0.7255 |

**ISC of character and action segments**

The higher ISC during violent action in comparison to character segments was further revealed in each of the 90-sec clips separately. The global ISC for both justified and unjustified violence showed that ISC was higher during action segments for each pair of movies as shown Supplementary Figure 2**.** The statistical analysis shows that the ISC was higher in action segments compared to character segments across all video clips.

**Supplementary Figure 2.** Global ISC during character and action segments**.** The boxplot shows the mean and the standard deviation The global ISC during action segments for both justified and unjustified movie violence were higher than the corresponding global ISC during character segments. (JC=justified character, JA=justified action, UC=unjustified character, UA= unjustified action, ** p<0.01, ***p<0.001)
